# The effect of the different restorations on fracture resistance of root-filled premolars

**DOI:** 10.1186/s12903-018-0663-7

**Published:** 2018-11-29

**Authors:** Hakan Göktürk, Emine Şirin Karaarslan, Elif Tekin, Bilal Hologlu, Işıl Sarıkaya

**Affiliations:** 1 Department of Endodontics, Bolu Abant İzzet Baysal University Faculty of Dentistry, Bolu, Turkey; 2 Department of Restorative Dentistry, Gaziosmanpasa University Faculty of Dentistry, Tokat, Turkey; 3 Department of Endodontics, Gaziosmanpasa University Faculty of Dentistry, Tokat, Turkey; 4 Department of Prosthodontics, Gaziosmanpasa University Faculty of Dentistry, 60100 Tokat, Turkey

**Keywords:** Endodontic treatment, Fracture resistance, Pre-impregnated glass-fibers

## Abstract

**Background:**

The study investigated the fracture resistance of root-filled maxillary premolars with class II cavities restored by different restorations.

**Methods:**

A total of 55 intact maxillary premolar teeth were included (*n* = 11). G1 as positive control group, 44 teeth underwent root canal treatment, and MOD cavities were prepared. (G2) no restoration, (G3) direct composite restoration, (G4) direct composite strengthened with buccal to lingual pre-impregnated glass-fibers and (G5) ceramic inlay restoration. After thermocycling, fracture resistance test was performed and fracture type was recorded. Data were analyzed with one-way ANOVA and Chisquare test.

**Results:**

The mean fracture resistance was as follows: G1 had the highest fracture resistance, G2 had the lowest (*p* < 0.05). There were no significant differences between the fracture resistance values of the groups that underwent different restorations (G3, G4, G5) (*p* > 0.05). According to fracture type, the groups showed similar results (*p* > 0.05). A significant level of unrestorable fracture was detected in G5 (ceramic inlay) (*p* < 0.05).

**Conclusions:**

All of the restoration techniques investigated herein increased the fracture strength of teeth; however, all of these values were lower than the fracture resistance of intact teeth. There were no significant differences between the fracture resistance values of the groups that underwent different restorations.

## Background

The fracture resistance of teeth can be decreased by cavities, trauma, erosion, aging, malocclusion, accidents, and caries [1]. In most cases, endodontically-treated teeth are more fragile due to non-conservative endodontic access cavity preparation, and removal of the arched roof of pulp chamber [2, 3]. The success of endodontic treatment is based on coronal restorations that support the remaining tooth structure, replace the tooth’s rigidity, and reduce coronal micro leakage [4–6].

Root-filled teeth are exposed to heavy masticatory loading forces. However, there is no consensus about the best restoration type of root-filled posterior teeth [3–7]. Treatment options to reinforce the residual tooth structure include composites, fiber reinforced composites, glass ionomer cement, and, if required, post and core restoration and crowns (full metal or porcelain-fused to metal crowns) [5, 8–10]. Direct resin bonded restorations are popular as they have sufficient aesthetic and physical properties, support weakened tooth structures, and can be applied during a single appointment. However, direct composite restorations have the disadvantage of polymerization shrinkage and stresses [11]. Polymerization shrinkage may be reduced by using flowable resin under the composite resin using soft-start irradiation protocols or using an incremental technique; however, these procedures require more chair time [2, 11].

The use of fiber-inserted composite restorations with different positions has been investigated with the goal of increasing the fracture strength of endodontically treated teeth [5, 7]. Belli et al. [2] reported that the use of polyethylene fibers under the composite restoration increases the fracture resistance of root canal filled teeth with mesio-occluso-distal (MOD) preparation. However, this procedure requires an additional step and time. This special fiber network can be made of glass, polyethylene, Kevlar, or carbon [12]. The reinforcing capacity of fibers is dependent on their orientation, on their adhesion to the resin, and on their impregnation with the resin [13]. Interlig (Angelus Ltd., Londrina, Brazil) is an example of pre-impregnated woven glass fiber composed of E-glass and bis-GMA. These pre-impregnated fibers have an aesthetic appearance and can tolerate 2–3 times more load than the hand-impregnated fibers [14]. However, there is limited knowledge about the effects of pre-impregnated fibers when used together with an extensive composite restoration on the fracture strength of root canal filled teeth [15].

An alternative to the restoration of endodontically-treated teeth includes adhesive ceramic inlays. These inlays are composed of feldspathic ceramic blocks that are either produced in a laboratory or milled chair-side via computer-assisted design/computer assisted machining (CAD/CAM) technology. It has been shown that patients treated with laboratory-processed ceramics require multiple visits, as these ceramics have worse fracture strength and structural homogeneity than feldspathic ceramic blocks [16]. Ceramic inlay blocks have high flexural strength, improved esthetics, optimized wear resistance [4, 17]. VITA ENAMIC (Vita Zahnfabrik, Bad Säckingen, Germany) is a hybrid ceramic block with a dual-network structure (86% ceramic network and 14% polymer network) [17]. According to manufacture VITA ENAMIC blocks have higher elasticity than traditional dental ceramics and better abrasion behavior than composites. However, indirect restorations require greater removal of tooth structure when compared with direct restorations [18].

The literature search revealed limited data on a comparison of fiber inserted composites and ceramic inlays on the fracture strength of root canal filled premolar teeth [9, 19]. Therefore, this in vitro study aimed to evaluate the strength required to fracture root canal filled maxillary premolars restored with different materials, including resin composite, glass-fiber reinforced resin composite, and indirect hybrid ceramic inlay in Class II adhesive cavity preparations. The null hypothesis was that there would be no difference between the materials used regarding the fracture resistance of root-filled teeth.

## Methods

Fifty five intact permanent human maxillary premolar teeth that were extracted for periodontal or orthodontic reasons were included in this study. Surface deposits were removed with scalers. The teeth were examined with transmitted light and stereomicroscopy (10×) for presence of abrasions, fractures, caries, and restorations; any tooth having one of these qualities was not included in the study, and was replaced with similar tooth. Each tooth was enumerated, and the mean weight of each tooth was calculated after weighing the tooth three times with an electronic balance (Kern ABJ, Kern & Sohn Gmbh, Ziegelei, Balingen, Germany) having an accuracy of 0.0001 g. Each tooth was randomly assigned to one of five groups; each group included 11 teeth, and the teeth were selected so that the mean tooth weight of each group was similar (Table 1). All teeth were stored in 0.1% thymol solution (Thymol, Merck KGaA, Darmstadt, Germany) until they were used.

**Table 1:** Distribution of tooth weights by groups

Groups	Mean (g) ± Standard Deviation	*p*
Group 1 (Positive control)	1.1645 ± 0.1008 ^A^	0.860
Group 2 (Negative control)	1.2889 ± 0.1049 ^A^	
Group 3 (Composite resin)	1.0600 ± 0.1318 ^A^	
Group 4 (Fiber+composite resin)	1.0643 ± 0.2257 ^A^	
Group 5 (Ceramic Inlay)	1.2363 ± 0.0985 ^A^	

Same superscript letter indicates statistically similar values (*p* > 0.05)

A silicon impression material (Impregum Soft; 3MESPE, Saint Paul, MN, USA) was used to coat the cementum surface of all roots up to 1 mm below the cemento-enamel-junction (CEJ); this was done to simulate 0.3-mm periodontal ligament space [4]. Then, the roots of the teeth were inserted parallel to the long axis of the teeth in self-curing acrylic resin.

The superimposed endodontic access cavities were prepared by the same experienced operator in all specimens [except for the positive control group (G1). The root canal preparation was performed with K files (apical size 40 up to 1 mm short of the apical foramen) using step back technique, and 1 mm was withdrawn after each file until size 70. The root canals were irrigated with 2 ml 5% NaOCl after each file change. A final rinse was performed with 5 ml 17% EDTA and 5 ml 5% NaOCl. The canals were dried with paper points and obturated with gutta-percha (Diadent Group International Inc., Chongju City, Korea) and 2Seal (VDW, Munich, Germany) using a cold lateral compaction technique. Finally, the excess root filling materials were removed and the cavity was cleaned. Pulp chambers were bonded by using self-etching bonding (Clearfil SE, Kuraray Noritake Dental Inc., Tokyo, Japan) and filled with flowable composite resin (Filtek Flow; 3 M ESPE Dental Products, St. Paul, USA). The coronal restorations were completed as indicated for each group.

**Fig. 1:**
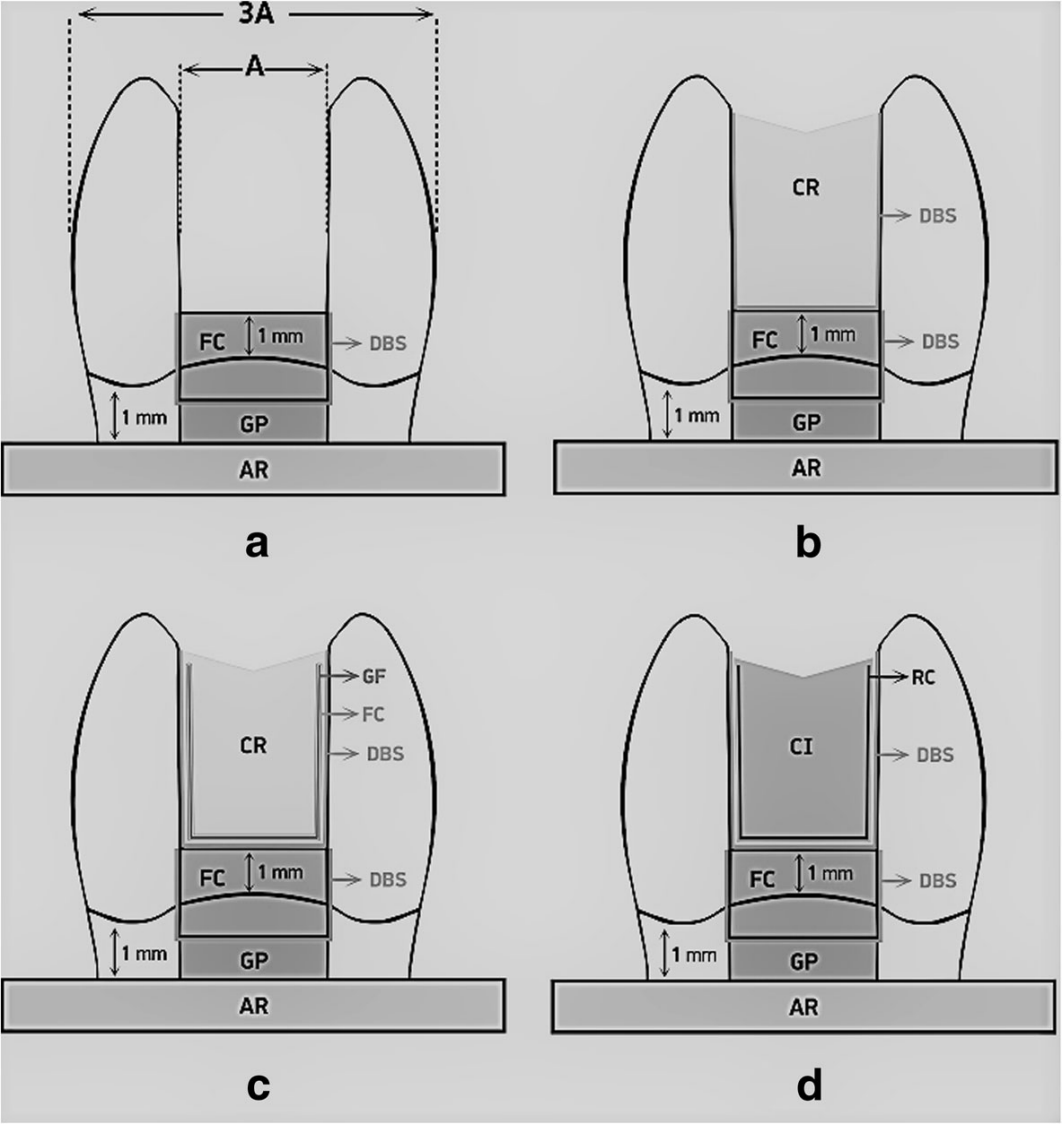
Schematic representation of the teeth and restorations. **a** Unrestored after endodontic treatment. **b** The restoration of teeth in G3 with composite resin following dentin bonding system application. **c** The restoration of teeth in G4 with composite resin following dentin bonding system application and glass fiber placement with flowable resin composite. **d** The restoration of teeth in G5 with cremic inlay following dentin bonding system and resin cement application. DBS, dentin bonding system; CR, composite resin; FC, flowable resin composite; GP, gutta-percha; AR, acrylic resin; GF, glass fiber; CI, ceramic inlay; RC, resin cement

For MOD cavity preparation, cylindrical diamond burs (Diatech, SwissDental Instruments, Heerbrugg, Switzerland) under air- water cooling was used in a high-speed headpiece (Kavo, Kaltenbach & Voigt GmbH, Biberach, Germany). The proximal box width of the cavities was one third of the bucco-lingual distance, and they extended into the pulp chamber. A digital caliper (Macrona, Simetri Teknik, İzmir, Turkey) with 0.01 mm accuracy was used to measure and obtain the standard cavity dimensions. The depth of the cavities was approximately 4 mm, and the cavities did not have proximal steps or a flat. After 3 preparations, the burs were replaced. All of the cavities were prepared so that the gingival cavosurface margin was located 1 mm above the CEJ. The lingual and buccal walls of the ceramic inlay cavities were prepared with tapered (6-degree) diamond burs using an electrical milling machine paralelometer (Paraskop M, BEGO, Germany) to obtain 6° divergence of the walls. Schematic representation of the teeth in each group was shown in Fig. 1 (a, b, c, d).

The five groups are listed according to the restoration type of access cavities as follows:

**Group 1 (G1; Positive control group):** This group included intact teeth without cavity preparation or root canal treatment.

**Group 2 (G2; Negative control):** Teeth in this group underwent the standardized MOD cavity preparation with superimposed endodontic access cavity as prepared by the same experienced operator in all specimens. All of the teeth underwent root canal treatment, but MOD cavity restoration was not performed.

**Group 3 (G3; Composite resin):** The self-etching bonding (Clearfil SE, Japan) was applied to the cavities according to manufacturers’ instructions. The polymerization of the bonding agent was performed with a light-emitting diode (LED-Elipar Free Light S10, 3M ESPE) at a minimum intensity of 1,200 mW/cm^2^. The cavity floor was covered with a 1 mm layer of flowable composite (Filtek Flow; 3M ESPE Dental Products, St. Paul, USA), and cavities were restored with a microhybrid composite resin (Filtek Z 250, 3M ESPE Dental Products, St. Paul, USA) using an oblique incremental technique (a maximum 2 mm of resin was placed in each turn, and each increment was light cured for 20 s).

**Fig. 2:**
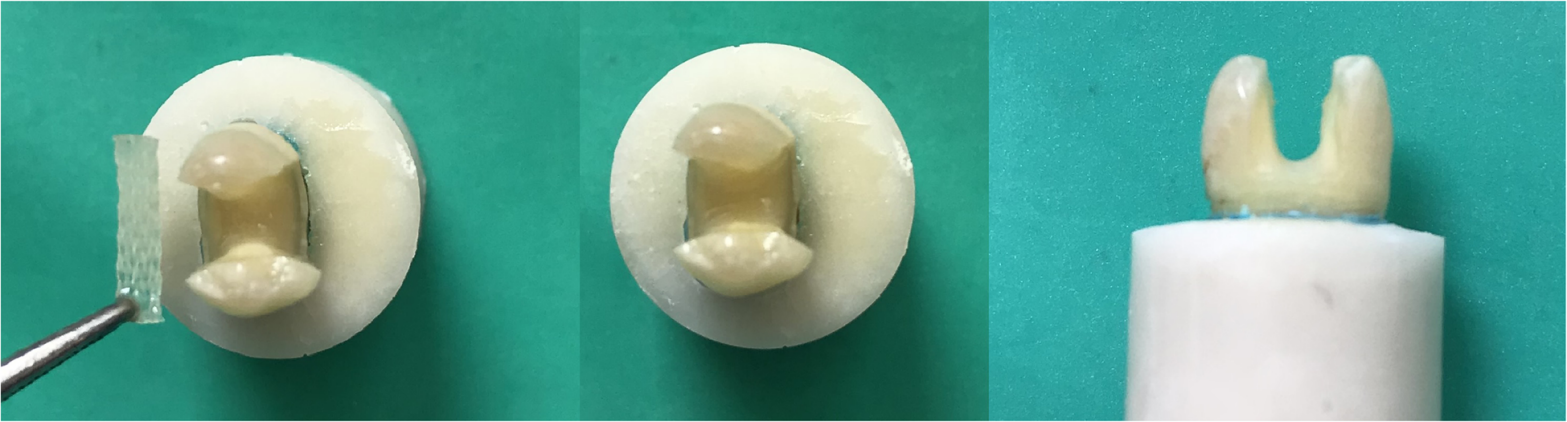
Fiber placement to the cavity

**Group 4 (G4; Glass-Fiber+composite resin):** The cavities were primed and bonded as in G3, and a thin layer of flowable resin composite was applied. A piece of Interlig (Angelus Ltd., Londrina, Brazil) (~8 mm long, 2 mm wide) was prepared and placed in the flowable resin from the buccal to lingual direction without touching the enamel margins (Fig. 2). After light-curing for 20 s with an LED, the remaining cavity space was restored with composite resin as in G3.

**Group 5 (G5; Ceramic inlay):** For this group, 11 inlays were fabricated by CAD/CAM device (CEREC 2; Sirona Dental Systems GmbH, Bensheim, Germany) using software (COS Crown 2.1; Sirona Dental Systems GmbH) from hybrid ceramic blocks (Vita Zahnfabrik, Bad Sackingen, Germany). Cavities were primed and bonded as in G3. The inner surfaces of inlays were prepared according to the manufacturer’s instructions. Then, the inner surfaces of inlays and cavity walls were coated with a thin layer of resin cement (Bisco Duolink, Bisco Inc., Schaumburg, USA). The inlays were inserted with a finger pressure, and excess cement was removed from the cavity margins using a scaler. The cement was light cured for 40 s each in the distal, mesial, and occlusal directions.

After the restorations in groups 3–5 were complete, they were finished and polished with discs (Soflex; 3 M ESPE, Seefeld, Germany). All restorations and preparations were performed by same dentist.

### Thermocycling and fracture resistance test

The specimens underwent thermocycling for 10,000 cycles between 5 and 55 °C with a dwell time of 30 s and a transfer time of 15 s (SD Mechatronik Thermocycler, SD Mechatronik GMBH, Westerham, Germany). After thermocycling, the specimens were placed into a Universal Testing Machine (AGS-X, Shimadzu, Kyoto, Japan) for a fracture resistance test, in which they were subjected to a compressive force at a crosshead speed of 1 mm/min with a round shaped modified bar with a diameter of 4 mm. A metal bar was positioned parallel to the long axes of the tooth and contracted against the occlusal surface of the restoration; the buccal and lingual cusps of tooth were used to apply the force. The maximum load necessary to fracture for each specimen was recorded in Newtons (N).

### Fracture mode

**Fig. 3:**
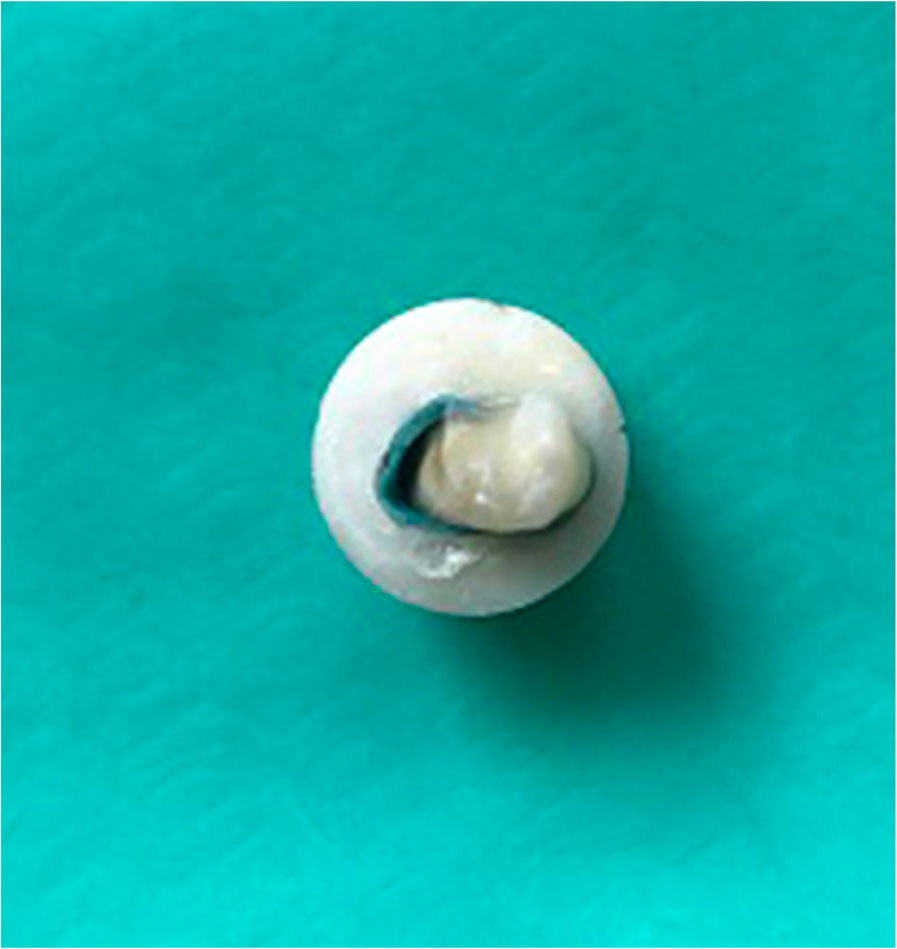
An example of unrestorable fracture for G3 (composite resin)

**Fig. 4:**
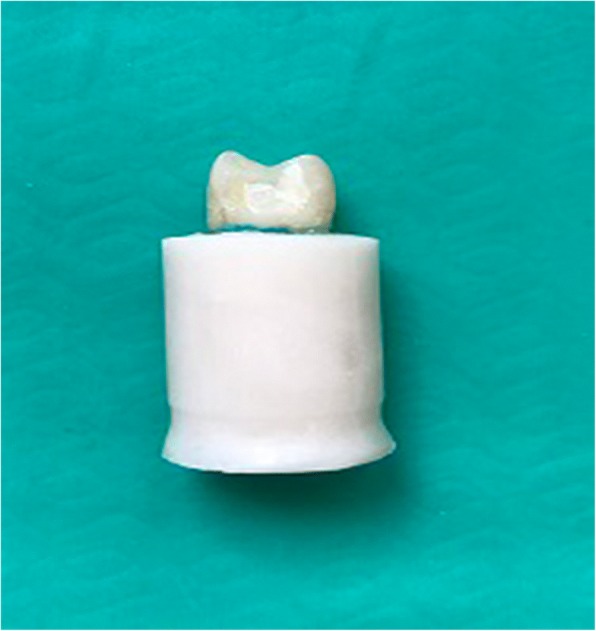
An example of restorable fracture for G4 (pre-impregnated glass fiber)

The specimens were examined under a stereomicroscope at 10× magnification. Fracture modes were classified according to Mohammadi et al. [19] as (i) restorable fractures (those extending up to 1 mm apical of the CEJ), or (ii) unrestorable fractures (those extending more than 1 mm apical of the CEJ) (Figs. 3 and 4).

### Statistical analysis

All analyses were performed using SPSS 19 (IBM SPSS Statistics 19, SPSS Inc., an IBM Co., Somers, NY). Data are expressed as mean ± standard deviation. A one-way ANOVA was used to compare the continuous data among groups. The Tukey HSD test was used for post-hoc comparisons between the pair-wise groups, while a Chisquare test (followed by Bonferroni correction) was used to compare the failure modes of each group. Values of *p* < 0.05 were considered significant.

## Results

### Fracture strength

The mean fracture resistance (N) for each group is shown in Table 2. According to the one-way ANOVA, teeth in G1 (positive control-intact teeth) had significantly higher fracture resistance values than the other groups, while the teeth in G2 (negative control-unrestored teeth) had the lowest values (*p* < 0.05). The fracture resistance of teeth with the G5 (ceramic inlay), the G4 (pre-impregnated glass fiber), and the G3 (composite resin) was statistically similar (*p* > 0.05). The fracture strength values increased in the teeth restored with G3 (composite resin), G4 (pre-impregnated glass fiber), and G5 (ceramic inlay) compared with unrestored teeth (G2) (*p* < 0.05).

**Table 2:** Distribution of fracture resistance according to groups

Groups	Mean (N) ± Standard Deviation	Minimum (N)	Maximum (N)
Group 1 (Positive control)	742.09 ± 245.45 ^A^	470.46	1192.30
Group 2 (Negative control)	192.12 ± 59.38 ^B^	120.97	278.18
Group 3 (Composite resin)	355.88 ± 103.94 ^C^	217.44	535.34
Group 4 (Fiber+composite resin)	367.12 ± 82.92 ^C^	228.67	529.41
Group 5 (Ceramic Inlay)	436.29 ± 69.56 ^C^	301.75	519.20

Same superscript letter indicates statistically similar values (*p* > 0.05)

### Fracture mode

**Fig. 5:**
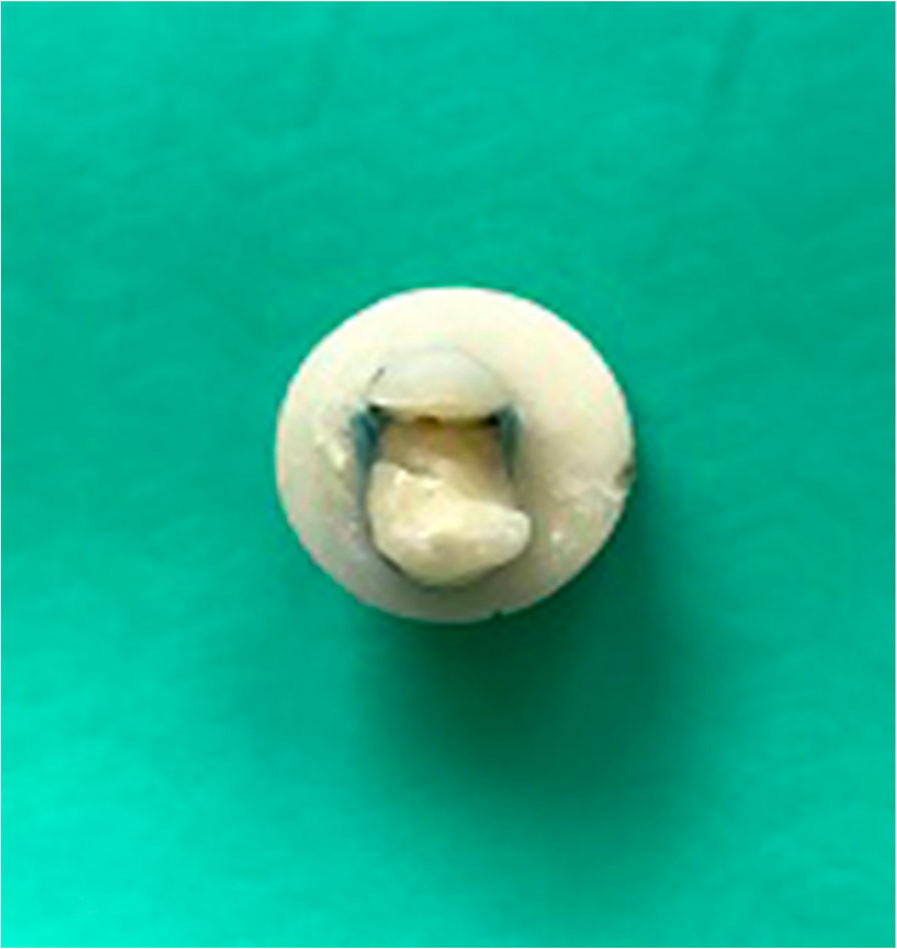
An example of unrestorable fracture for G5 (ceramic inlay)

Table 3 shows the fracture modes and distributions of each group. The most restorable fractures were observed in G1 (intact teeth). There were no statistically significant difference according to fracture modes in G2 (unrestored teeth), G3 (composite resin), and G4 (pre-impregnated glass fiber) (*p* > 0.05). A significant level of unrestorable fracture was detected in G5 (ceramic inlay) (*p* < 0.05) (Fig. 5). There was a significant difference between G1 and G5 and between G1 and G4 according to fracture type distribution (*p* < 0.05).

**Table 3:** Distribution of fracture modes according to groups

Groups	Fracture Modes		*p*
	Unrestorable	Restorable	
Group 1 (Positive control)^A^	0 (0)^a^	11 (34.4)^b^	0.004
Group 2 (Negative control)^AB^	5 (21.7)^a^	6 (18.8)^a^	
Group 3 (Composite resin)^AB^	3 (13)^a^	8 (25)^a^	
Group 4 (Fiber+composite resin)^B^	7 (30.4)^a^	4 (12.5)^a^	
Group 5 (Ceramic Inlay)^B^	8 (34.8)^a^	3 (9.4)^b^	
Total	23 (100)	32 (100)	

Same superscript uppercase letters indicate statistically similar values within each column (*p* > 0.05). Same superscript lowercase letters indicate statistically similar values within each row (*p* > 0.05)

## Discussion

This in vitro study investigated the fracture resistance of root-filled maxillary premolars restored with different materials, including direct resin composite, glass-fiber reinforced resin composite, and indirect hybrid ceramic inlay, in Class II adhesive cavity preparations. The results of this study supported the null hypothesis, which stated that the different materials used in the restorations would not affect the fracture strength. Although the tested restoration techniques increased the fracture strength of teeth, there were no significant differences among the fracture resistance values of direct composite, pre-impregnated glass-fiber reinforced composite, and hybrid ceramic inlay restorations.

In the studies, it was reported that the mesiodistal (MD) and buccolingual (BL) dimensions, as well as the lengths of the teeth, were considered in the standardization of samples [9, 20]. In another study, Yasa et al. [21] reported that a direct correlation was observed between the fracture resistance and the weight of the tooth, rather than their MD and BL dimensions. In the current study, we created groups with teeth having similar weights. The weights of the teeth were considered in the standardization of samples.

The periodontal ligament, which is approximately 0.1–0.3 mm thick [22], absorbs occlusal loads. When an excessive force is exerted on the tooth, the periodontal fiber compression provides an important support to the tooth [23]. In order to more closely simulate the clinical situations and to create the absorption of chewing forces by bone and periodontal ligaments in vitro situations, many studies discuss the importance of simulated periodontal ligament [4, 20]. However, Marchionatti et al. [24] reported that the artificial periodontal ligament has no effect on the fracture or bond strength. In the current study, the roots were surrounded with polyvinyl siloxane up to 1 mm below the CEJ to simulate 0.3-mm periodontal ligament space.

The structural strength of dentin tissue depends on the integrity and quality of its anatomic structure; therefore, it is important to protect the sound dentin tissue that retains and supports the restoration in cavity preparations [25]. The quantity of the residual sound dentin after cavity preparation is the main factor determining the fracture resistance of the tooth [26]. It has been reported that the fracture strength significantly decreases in large cavity preparations and/ or in the teeth with large tissue losses. MOD cavity preparation and root canal treatment reduce the fracture strength of the tooth due to great removal of the tooth structure [2, 4]. It has been stated that intact maxillary premolars can be fractured at a load of 1121 N, and intact molars can be fractured at a load of approximately 2500 N. Additionally it has been shown that the fracture resistance of teeth with MOD cavities decreased by 54% compared to their original resistances [27]. Therefore, in this vitro study, premolar MOD cavities were prepared in order to compare the reinforcing effects of different restorative materials in the worst dental cases.

Residual dental tissue must be reinforced in order to support the cavity with a restorative material [4]. It is known that the success of endodontic treatment depends on the quality of the coronal restoration [6]. It has been reported that teeth with crown rehabilitation had a six-fold higher rate of success than teeth that were directly restored [8]. However, our current study utilized more conservative approaches in order to preserve sound tissue.

Adhesive restorations are better able to distribute functional stresses throughout the tooth and restorative material interface, and they also have the potential to support the fragile dental structure [28]. The composite restorations that bond directly to dentin enhance the durability of unsupported tooth structures [3]. However, composite resins may undergo polymerisation shrinkage during curing [29]. Therefore, an incremental technique is recommended in order to decrease the cuspal deflection produced by polymerization contraction stresses [30]. An alternative method for increasing the fracture strength of root canal filled teeth is the insertion of fibers, which are increasingly being used for the reinforcement of polymer-based dental materials. Many studies have investigated the use of ultra-high-molecular-weight polyethylene fibers, which have an ultra-high elastic modulus [4, 5]. In previous studies, polyethylene fibers with a low flexural modulus and high modulus of elasticity were used to transfer stress along the restoration and tooth surfaces in endodontically treated teeth. These studies reported that polyethylene ribbond fibers in endodontically-treated molar teeth with MOD preparations increased the stiffness of the restored tooth [2, 3]. However, Cobankara et al. reported there were no significant differences in the fracture strength of molar teeth restorated with composite resin or fiber-inserted composite resin [4]. Similarly in the present study, when compared to composite resin, fiber-insered composite resin improved the fracture strength of premolar teeth; however, this improvement was not significant.

The presence of glass-fibers in the resin composite could alter the elastic modulus of the material itself, thus modifying the stress distribution and transmission to residual cavity walls [15]. In their study, Nicola et al. [15] evaluated the fracture strength of root canal filled mandibular first molars restored with different restorative systems (direct composite restoration, fiber-post supported composite restoration, MD glass-fiber reinforced composite restoration, buccal-oral glass-fiber reinforced composite restoration). They reported that for the direct restoration of root canal filled molars, composite resins strengthened with glass-fibers or fiber posts can enhance fracture strength. In the current study, pre-impregnated glass fibers composed of E-glass were placed from buccal to lingual direction without touching the enamel margins. We found that the fracture strength values of the teeth restored with resin composite, pre-impregnated glass fibers, and ceramic inlay (G3, G4, and G5) were higher than those of the unrestored group (G2). However, there were no significant differences between the fracture resistance values of the G3, G4, and G5 groups. In the study by Nicola et al. [15], there were no significant differences among experimental groups; however, the buccal-oral pre-impregnated glass-fiber reinforced composite restoration showed the highest fracture strength of the groups (except for intact teeth).

After treatment, restorable fractures in the teeth have a better prognosis than do unrestorable fractures. Similar to previous studies, in the current study, we found more restorable fractures in the intact teeth than in those teeth that were restored [19, 26]. This might be related to the regular distribution in the load applied to the tooth along the intact dental structures [26]. In the G5 (ceramic inlay), untreatable fractures were detected at a significantly higher level than those of the other groups. Results of the current study indicate that as teeth are restored with more rigid materials, the incidence of unrestorable fractures increases. However, further investigations are needed to reassess these results. This study is limited in that the oral environment cannot be fully reflected, and that a chewing simulator device was not used.

Dentin is the major structural component of the tooth. With age, the micro-structure of the dentin changes, mineral content increases and sclerosis begins. Previous studies report that fracture resistance of old dentin is lower that young dentin [31, 32]. Therefore, one of the limitations of the study was that age distribution among the groups was not considered. The large standard deviations on the fracture resistance value of the teeth might be attributed to age changes in the dentin.

Cuspal coverage with direct or indirect composite restoration is an alternative, conservative and one-appointment complementary treatment option in large cavities. Resin composite, amalgam or combined composite-amalgam was used to cuspal coverage [33]. Fracture resistance of cuspal coverage of endodontically treated maxillary premolars with combined composite-amalgam compared to other techniques. In previous studies, it was reported that cuspal coverage increases the fracture resistance of weakened endodontically treated teeth when compared to teeth restored without cusp coverage [34, 35]. Full metal-ceramic crowns is another treatment option, however, it causes a significant reduction in tooth structure and brings with increased cost and time loss [36].

## Conclusion

Under the conditions of this in vitro study, it was concluded that:

1. The tested restoration materials enhanced the fracture strength of teeth, but these values were fairly lower than the fracture strength of intact teeth.
2. There were no significant differences between the fracture resistance values of the groups that underwent different restorations.
3. When the fracture modes were considered, the groups showed similar results. However, G5 (ceramic inlay) showed a significantly unrestorable fracture.

## Abbreviations

BL: Bucco-lingual
CAD/CAM: Computer-assisted design/computer assisted machining
CEJ: Cemento-enamel-junction
LED: Light-emitting diode
MD: Mesio-distal
MOD: Mesio-occluso-distal
N: Newton
